# Study on Spatial Effects of Influencing Factors and Zoning Strategies for PM_2.5_ and CO_2_ Synergistic Reduction

**DOI:** 10.3390/toxics12070498

**Published:** 2024-07-09

**Authors:** Zimu Jia, Shida Sun, Deming Zhao, Yu Bo, Zifa Wang

**Affiliations:** 1 State Key Laboratory of Atmospheric Boundary Layer Physics and Atmospheric Chemistry, Institute of Atmospheric Physics, Chinese Academy of Sciences, Beijing 100029, China; jiazimu@mail.iap.ac.cn (Z.J.); zifawang@mail.iap.ac.cn (Z.W.); 2 Ministry of Education Key Laboratory for Earth System Modeling, Department of Earth System Science, Tsinghua University, Beijing 100084, China; sunshida.2021@tsinghua.org.cn; 3 Key Laboratory of Regional Climate and Environment for Temperate East Asia, Institute of Atmospheric Physics, Chinese Academy of Sciences, Beijing 100029, China; zhaodm@tea.ac.cn

**Keywords:** synergistic reduction, evaluation index, influencing factors, spatial effects, zoning strategies

## Abstract

China has identified the synergistic reduction of pollution and carbon emissions as a crit ical component of its environmental protection and climate mitigation efforts. An assessment of this synergy can provide clarity on the strategic management of both air pollution and carbon emissions. Due to the extensive regional differences in China, the spatial effects of influencing factors on this synergy exhibit variation across different provinces. In this study, the reduction indexes of PM_2.5_ and CO_2_ were calculated based on their reduction bases, reduction efforts, and reduction stabilities across provinces. Then, the synergistic reduction effect was assessed using an exponential function with the PM_2.5_ reduction index as the base and the CO_2_ reduction index as the exponent. Next, the MGWR model was applied in order to analyze the influencing factors of the synergistic reduction effect, considering natural settings, socioeconomic conditions, and external emission impacts. Finally, the k-means clustering method was utilized to classify provinces into different categories based on the degree of impact of each influencing factor. The results indicated that air circulation, vegetation, tertiary industry ratio, and emission reduction efficiency are major impact indicators that have a positive effect. The topography and emissions from neighboring provinces have a statistically significant negative impact. The spatial influences of different factors exhibit a distribution trend characterized by a high-high cluster and a low-low cluster. A total of 31 provinces are divided into three categories, and suggestions on the corresponding category are proposed, to provide a scientific reference to the synergistic reduction of PM_2.5_ and CO_2_.

## 1. Introduction

China is facing the dual pressures of reducing air pollution and carbon emissions [1,2]. In response to this challenge, a series of policies have been implemented by the Chinese government. The Air Pollution Prevention and Control Action Plan was issued in 2013, followed by the Three-Year Action Plan for Winning the Blue Sky Defense Battle in 2018, aiming to improve nationwide air quality [3]. Meanwhile, China has acknowledged its commitment to reducing CO_2_ emissions to combat global warming, as stipulated in the Paris Agreement. For example, China has formally committed to “double carbon” targets, striving to achieve a carbon peak by 2030 and carbon neutrality by 2060 [4]. For a long time, air pollution and carbon emissions were treated as two separate issues with different strategies [5]. Actually, air pollutants and CO_2_ are derived from similar sources and are released simultaneously, implying that effective management of both air pollution and carbon emissions can result in a synergistic reduction [6]. In 2022, the Chinese government announced the Implementation Plan for Synergic Efficiency in Reducing Pollution and Carbon, explicitly promoting a strategy for the synergistic reduction of air pollutants and CO_2_ emissions. Given China’s extensive regional differences in terms of meteorological conditions, topography, population density, economic growth, energy structure, energy efficiency, technological innovation, etc., the directions and mechanisms of the impacts of different factors on the synergistic reduction may vary between provinces. Combining various drivers with positive and negative effects can reveal the spatial disparity of synergistic reduction effects at the provincial level in China, thereby avoiding a one-size-fits-all policy.

Since air pollution and greenhouse gas emissions have the same origin and spatial consistency, they should be assessed together rather than separately [7,8]. Existing studies have confirmed that there is a synergistic reduction between air pollutants and greenhouse gases, and often focus on the magnitude of the decrease in emissions [9,10,11]. It is feasible to quantify the degree of synergistic reduction by constructing and calculating an indicator or index. Ref. [12] captured the damage value weights of air pollutants and CO_2_ and combined all the emissions as an air pollutant equivalence (AP_eq_) to reflect the co-control effect. The co-reduction coordinate system and cross-elasticity are also commonly used for evaluating synergies [13,14,15]. Ref. [13] analyzed the co-benefits of reducing air pollutants and CO_2_ emissions from electric private cars, taxis, and buses by using the co-control coordinate system and pollutant reduction cross-elasticity. Ref. [15] applied different cross-elasticities to quantitatively assess the co-benefits of lowering CO_2_ and atmospheric pollutants of various measures in the Air Pollution Prevention and Control Action Plan in the Jing-Jin-Ji region of China. These two methods can show the positive and negative effects of synergies based on the reductions in air pollutants and CO_2_. Other scholars analyzed the effect with models such as life cycle assessment (LCA) [16], greenhouse gas and air pollution interactions and synergies (GAINS) [17,18], and the long-range energy alternatives planning system (LEAP) [19,20]. Much of the literature focuses on the synergies between air pollutants and CO_2_ in terms of the quantities of reductions. Actually, according to China’s 2030 carbon peaking plan, carbon emissions are expected to continue rising in the coming years [21], leading to the carbon reduction that is encouraged but not compulsory. Therefore, negative synergies may be a prevalent phenomenon, meaning a decrease in air pollutants with a concomitant increase in carbon emissions. Additionally, extensive research observes the synergistic effect from a single perspective by examining the actual emission reductions, but ignores the contribution of emissions at the initial time point and how the reduction stability changes over time. The actual emission reductions reflect the direct effectiveness of a region, but if the initial emissions are disregarded, it tends to exaggerate the reduction efforts of the region and weaken the need for emission reductions. Furthermore, a consistent decrease in emission reduction levels or pollution concentrations within a certain area can also suggest that reduction policies and measures have played a stabilizing role without being ineffective.

The emissions of air pollutants and CO_2_ are closely related to fossil fuel consumption. The increase in energy demand during rapid economic development is a driver of air pollution and CO_2_ emissions [22,23]. Burning coal produces about 70% of the CO_2_ released into the air in China [24], and more than 80% of PM_2.5_ pollution comes from the coal used in the electricity and metal industries [25]. Therefore, coal combustion is considered an important factor in air pollution and CO_2_ emissions. Previous studies have also revealed that the expansion of PM_2.5_ and CO_2_ emissions is attributed to economic growth [26,27,28]. Particularly, the secondary industry in China includes energy-intensive and high-polluting sectors, indicating that the augmentation of the proportion of secondary industry contributes to air pollution and CO_2_ emissions [26,27]. Ref. [29] found that several factors—such as urbanization, industry, economy, energy, and so on—influenced the synergistic degree of air pollution control and CO_2_ reduction, thereby causing regional disparities. Additionally, the synergistic reduction of air pollutants and CO_2_ can be achieved through technological innovation [30]. For example, digital technologies have been used to monitor and analyze energy consumption in real time by many companies, enabling more efficient minimization of air pollution and carbon emissions [31,32]. Several researchers believe that the implementation of relevant policies for emission reduction is an important reason for the change in synergistic benefits, such as the Air Pollution Prevention and Control Action Plan [3], environmental protection tax [33], carbon pricing policy [34], low-carbon city pilot [35], etc. Generally, the existing studies focus on exploring the impacts of socioeconomic influencing factors on the synergistic reduction of air pollutants and CO_2_, but few investigate how natural factors such as meteorological and topographical conditions influence this synergy. In addition, research on influencing factors has only been carried out in a certain region, which fails to deal with the impact of emissions from adjacent areas.

In this study, an evaluation index was developed to comprehensively reflect the effectiveness of synergistic PM_2.5_ and CO_2_ reductions in each region. Compared with previous studies, the index integrates and quantifies PM_2.5_ emissions, PM_2.5_ annual average concentration, and CO_2_ emission intensity in terms of the reduction basis, reduction efforts, and reduction stability. In addition, this study thoroughly examines the impacts of the natural environment, socioeconomic conditions, and external emission factors on the spatial variations in the effectiveness of synergistic emission reductions across different areas. The findings can serve as a guide for the future implementation of tailored emission reduction policies.

The rest of the paper is organized as follows. Section 2 describes the methodology in detail, which mainly consists of the multiscale geographically weighted regression (MGWR) model and the k-means clustering method. In addition, data sources are represented in this section. Section 3 presents the outcomes of the spatial distribution patterns, including constructed indexes, coefficients of affecting factors, and zoning information. Discussions based on the results and conclusions are provided in Section 4 and Section 5, respectively.

## 2. Materials and Methods

### 2.1. Data Collection

The study covered 31 provinces in China, except for Hong Kong, Macau, and Taiwan, due to insufficient data. PM_2.5_ and CO_2_ emission data were taken from the Multi-Resolution Emission Inventory for China (http://meicmodel.org.cn (accessed on 5 July 2024)), established by Tsinghua University. The annual average concentration of PM_2.5_ was derived from reports on the state of the ecology and environment in 31 provinces, published by the provincial Department of Ecology and Environment. Boundary layer height and wind speed were collected from the ERA5 database (https://cds.climate.copernicus.eu (accessed on 5 July 2024)). The information on the Digital Elevation Model (DEM) and Normalized Difference Vegetation Index (NDVI) was obtained from the Resource and Environment Science and Data Center (https://www.resdc.cn (accessed on 5 July 2024)). Data on economic development, energy consumption, and forest cover were obtained from the China Statistical Yearbook and the China Energy Statistical Yearbook, released by the National Bureau of Statistics of China. All the data contain values spanning from 2016 to 2020.

### 2.2. Evaluation Index for PM_2.5_ and CO_2_ Reductions

This study evaluates the relative effects of PM_2.5_ and CO_2_ reductions on different emitting entities during a given period from three perspectives: the targeted reduction, the actual reduction, and the fluctuation. PM_2.5_ reduction includes the decrease in emissions and annual concentration, and CO_2_ reduction refers to the decrease in emission intensity. The target reduction is the difference between the emission result of an emitting entity and the corresponding standard in the starting year, which mainly assesses the basis for reductions in the emitting entity. Corresponding standards can be explicitly issued by the state—for example, ambient air quality standards, or those that are difficult to specify at this stage but are considered theoretically necessary to achieve, e.g., zero emissions. The actual reduction refers to the difference in emission results between the starting year and the target year, and is used to evaluate reduction efforts. The fluctuation value means the disparity between the sum of the absolute value of the actual reduction over a specified period, such as one year or one month, and the absolute value of the actual reduction over the entire period. The stability of reductions by emitting entities is primarily measured by the fluctuation value.

Since PM_2.5_ and CO_2_ reductions involve variables with different scales and units, they need to be converted to the same range for comparison and analysis. This will eliminate the discrepancy between scale and unit. The Sigmoid function will be able to map a number to the interval (0,1). Compared to the Min-Max Scaling normalization method, the Sigmoid function is not affected by extreme values and is more resistant to interference. However, the Sigmoid function belongs to the one with a saturated S-curve at both ends, and when the values on both sides of the definition domain exceed a certain range, the Sigmoid function curve tends to flatten. Therefore, the Sigmoid function input values need to be scaled by the same proportion. In this study, the Sigmoid function was used to calculate the targeted reduction index, the actual reduction index, and the fluctuation rate, based on which the reduction index was obtained. The equations are as follows:

(1) $$I_{TR}=\frac{1}{1+e^{\left(x_{1}-x_{s}\right)/m}}$$

(2) $$I_{AR}=\frac{1}{1+e^{\left(x_{n}-x_{1}\right)/m}}$$

(3) $$FR=\frac{1}{1+e^{\left(\left|x_{n}-x_{1}\right|-\sum_{i=2}^{n}\left|x_{n}-x_{n-1}\right|\right)/m}}$$

(4) $$I_{R}=I_{TR}+I_{AR}-FR$$

where *I_TR_* is the targeted reduction index; *I_AR_* is the actual reduction index; *FR* is the fluctuation rate; *I_R_* is the reduction index; *x_s_* is the corresponding standard value, *x_s_* = 35 μg/m^3^ (for PM_2.5_ annual average concentration) or *x_s_* = 0 (for PM_2.5_ emissions and CO_2_ emission intensity); *x*_1_ is the actual value in the starting year; *x_n_* is the actual value in the target year; *m* is the scaling factor, *m* = 10 (for PM_2.5_ emissions and annual average concentration) or *m* = 1 (for CO_2_ emission intensity). In the study, the starting year is 2016 and the target year is 2020.

During this study, the evaluation index for PM_2.5_ and CO_2_ reductions was calculated in the form of an exponential function. For the exponential function *y* = *a^x^*, when 0 < *a* < 1 and *x* ≥ 0, the exponential function has the following properties: (1) *y* takes on the range of (0,1]; (2) the function monotonically decreases; and (3) if *x* is the same, *y* increases as *a* increases. In this study, the PM_2.5_ reduction index and CO_2_ reduction index have values in the range of [0,1] basically, which can be used as the base and exponent of the exponential function to convert the final evaluation result to the (0,1] interval. The reduction index is a positive indicator—the greater the number, the better. However, the decreasing monotonicity of the function implies that the smaller the value at the exponential position, the better. Therefore, it is necessary to transform the PM_2.5_ reduction index or CO_2_ reduction index located at the exponential position. According to the property (3), the base and the function value keep the same increase and decrease. For the reduction index at the base position, it can be intuitively reflected as a positive indicator without the need for formal transformation. Compared with CO_2_ emissions, PM_2.5_ emissions have a more direct and intense impact on public health and socioeconomic development. China has been carrying out nationwide PM_2.5_ pollution control since 2013, and it was not until 2020, when the “dual carbon” target was proposed, that CO_2_ emission reduction was elevated to the same importance as PM_2.5_ pollution control. Therefore, the PM_2.5_ reduction index was applied as the base and the CO_2_ reduction index as the exponent. Since PM_2.5_ reduction includes emissions and annual concentration, the PM_2.5_ reduction index was calculated by using the exponential function, with the PM_2.5_ concentration reduction index as the base and the PM_2.5_ emission reduction index as the exponent. The equations are as follows:

(5) $$I_{R\_{\mathrm{PM}}_{2.5}}={I_{RC}}^{\left(1-I_{RE}\right)}$$

(6) $$EIPC={I_{R\_{\mathrm{PM}}_{2.5}}}^{\left(1-I_{R\_{\mathrm{CO}}_{2}}\right)}$$

where $I_{R\_PM_{2.5}}$ is the PM_2.5_ reduction index; $I_{R\_{\mathrm{CO}}_{2.}}$ is the CO_2_ reduction index; *I_RC_* is the PM_2.5_ concentration reduction index; *I_RE_* is the PM_2.5_ emission reduction index; *EIPC* is the evaluation index for PM_2.5_ and CO_2_ reductions.

### 2.3. Multiscale Geographically Weighted Regression

Ordinary least squares (OLS) is a global regression model that overlooks differences in variables due to spatial locations. Geographically weighted regression (GWR) extends OLS by assuming that the relationships between the response variable and explanatory variables change over space. Hence, GWR estimates a separate model with local parameters for each geographical location [36]. The bandwidth of an explanatory variable indicates its spatial scale of influence. GWR considers a fixed bandwidth for all parameters. Actually, a fixed bandwidth is not valid when phenomena involve multiple spatial processes with diverse explanatory variables [37]. MGWR allows relationships between the response variable and explanatory variables to change at different spatial scales [38,39]. This multi-bandwidth approach produces a more accurate result for the regression, representing spatial phenomena in the real world. The equation is as follows:

(7) $$y_{i}=\beta_{0}(u_{i},v_{i})+\sum_{k=1}^{m}\beta_{bwk}(u_{i},v_{i})x_{ik}+\varepsilon_{i}$$

where *u_i_* and *v_i_* are the coordinates of each explanatory variable at position *i*; *β*_0_(*u_i_*, *v_i_*) is the intercept value at position *i*; *bwk* is the bandwidth of the *k*th explanatory variable at position *i*; *β_bwk_*(*u_i_*, *v_i_*) is the regression coefficient of the *k*th explanatory variable at position *i*; *m* is the number of explanatory variables; *ε_i_* is the error value.

In this study, EIPC was utilized as the response variable for MGWR. According to the previous studies [27,28,29,30] and the data availability, explanatory variables comprised air circulation level (ACL), topographical relief level (TRL), NDVI, forest coverage rate (FCR), stock volume of forest (SVF), gross domestic product (GDP), proportion of the tertiary industry (PTI), emission reduction efficiency (ERE), PM_2.5_ emissions from neighboring provinces (ENP), and net electricity demand from other provinces (NED). The variables mentioned in the MGWR model were computed as the average values across the years 2016 to 2020. ACL is constructed by multiplying boundary layer height by average wind speed, which determines the dispersion of pollutants. For two places with identical emissions levels, the one with a higher ACL experiences lower PM_2.5_ pollution [40]. TRL is the average of the difference between the DEM of each city boundary in a province and the average DEM of the plains within that city. A greater TRL indicates that the province may possess more cities of the basin type, which can result in the accumulation of pollutants. Vegetation plays an important role in absorbing carbon dioxide and reducing the concentration of particles. Its growth stage and health level also affect its ability to eliminate CO_2_ and particles [41]. NDVI is often used to assess the spatial distribution and growth status of vegetation. However, NDVI is processed using nonlinear stretching in the calculation process, resulting in a low sensitivity to highly vegetated areas. Combining other data can provide more comprehensive vegetation information. Forests, as one of the most important components of ecosystems, are vital for carbon sequestration and particle removal compared to other vegetation systems, such as grasslands. FCR is the ratio of forest area to total land area within the administrative region, and SVF refers to the total stock volume of tree trunk timber in a given forest area. These two indicators reflect, to some extent, forest resources in terms of quantity and quality. China is currently in the industrialization stage and relies on substantial energy inputs, resulting in a certain positive correlation between economic development and air pollution and carbon emissions. Increased PTI contributes to improved energy efficiency and reduced energy use, lowering pollutants and carbon emissions from industrial production [30]. One region is susceptible to PM_2.5_ emissions from neighboring provinces due to atmospheric transport. NED is the difference between annual electricity consumption and annual electricity generation. A positive value indicates that electricity needs to be imported from other provinces, resulting in the transfer of air pollution and carbon emissions from electricity-consuming to electricity-producing areas. ERE suggests the efficiencies of technologies and management levels in the process of PM_2.5_ and CO_2_ emission reductions while keeping all inputs constant. In this paper, ERE was calculated by adopting the data envelopment analysis (DEA) approach. DEA does not require prior assumptions of functional relationships between inputs and outputs, and can avoid man-made subjectivity for parameter weighting [42]. This model has been extensively utilized to assess energy efficiency and CO_2_ emission efficiency [43]. In the developed DEA model, PM_2.5_ and CO_2_ emission reductions were taken as outputs, while coal consumption for thermal power, coal consumption for industry, coal consumption for residents, and completed investment in projects for treating waste gases were viewed as inputs.

### 2.4. K-Means Clustering

The k-means clustering method involves an unsupervised recognizable pattern in which the objects prepared to be classified in a data set are efficiently computed and categorized into proper groups. The classification of objects is based on several types of indicator, which are appropriate for describing the characteristics of objects. In this study, the regression coefficients for different explanatory variables of each province were generated by the constructed MGWR model. These regression coefficients are the classification criteria of the k-means clustering. Provinces are categorized into corresponding categories to identify the similarities and distinctions observed among diverse groups.

As the k-means clustering method requires the user to prespecify the number of clusters, it is indispensable for measuring the clustering quality. The silhouette coefficient combines both cohesion and separation, and is rather independent of the number of clusters. The optimal clustering number can be obtained by calculating the average silhouette coefficient of all the provinces. For one cluster with k categories, the average silhouette coefficient refers to the average of the silhouette coefficients of provinces belonging to the cluster [44].

## 3. Results

### 3.1. Spatial Characteristics of Reduction Indexes

**Figure 1:**
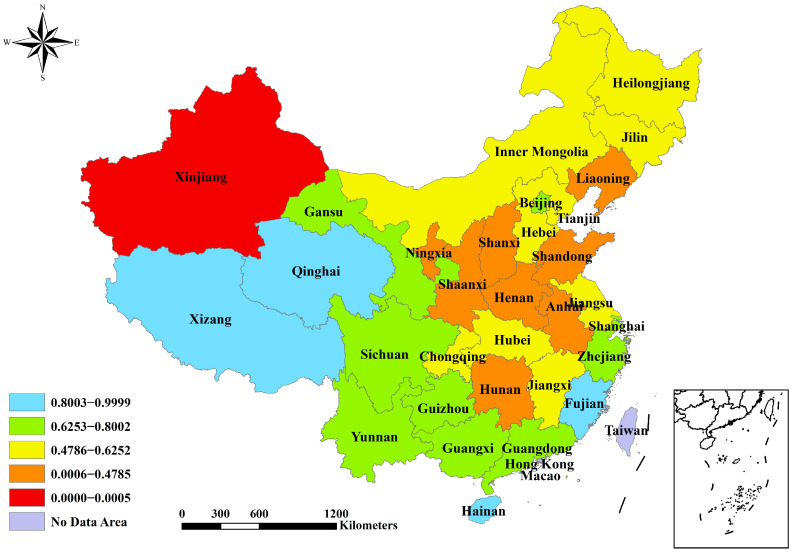
Spatial characteristics of PM_2.5_ reduction index 2016–2020.

**Figure 2:**
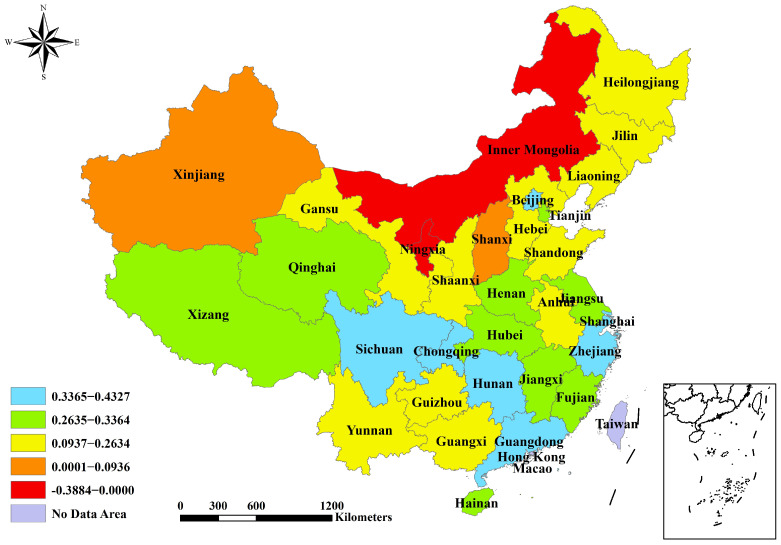
Spatial characteristics of CO_2_ reduction index 2016–2020.

**Figure 3:**
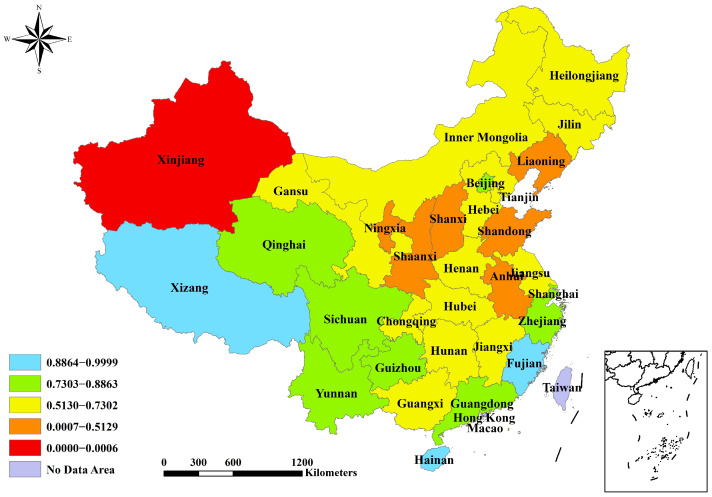
Spatial characteristics of evaluation index for PM_2.5_ and CO_2_ reductions from 2016–2020.

The reduction index values have the potential to range from 0 to 1. A province’s effectiveness in reduction increases as the value approaches 1. The values were categorized using the natural breakpoint classification method in the ArcGIS 10.6 software(Environmental Systems Research Institute, Redlands, CA, USA). For the PM_2.5_ reduction index, Xinjiang has the lowest value and is categorized separately (Figure 1). Four provinces have a value greater than 0.81 and are deemed to have relatively better effectiveness in controlling PM_2.5_, namely Xizang (0.9999), Hainan (0.9820), Fujian (0.8724), and Qinghai (0.8453) (Table 1 and Figure 1). The values are generally higher in the provinces of the southwestern and southern regions compared to those in the provinces of the northern and central areas. For the CO_2_ reduction index, the results for Ningxia and Inner Mongolia are below zero because of the significant fluctuations in their annual falls of CO_2_ emission intensity (Table 1). Seven provinces have a rating above 0.34 and are considered more successful in managing CO_2_ emission intensity, namely Beijing (0.4327), Shanghai (0.3848), Guangdong (0.3674), Chongqing (0.3533), Zhejiang (0.3532), Sichuan (0.3531), and Hunan (0.3485) (Table 1 and Figure 2). The southern provinces outperform the northern provinces in reducing CO_2_ emission intensity. For the evaluation index of PM_2.5_ and CO_2_ reductions, Xinjiang is the region with the lowest value (0.0006) and is classified as its own category. Tibet, Hainan, and Fujian obtained scores exceeding 0.9 and are recognized as top performers (Table 1 and Figure 3). Furthermore, approximately half of the provinces achieved moderate outcomes in terms of synergistic reductions in PM_2.5_ and CO_2_, with scores between 0.57 and 0.74.

### 3.2. Spatial Characteristics of Coefficients in MGWR

Table 2 compares the fitting results of the OLS, GWR, and MGWR models. The outputs suggest that the MGWR model outperforms OLS and GWR, with lower AICc and higher R^2^ and Adj-R^2^. The AICc, R^2^, and Adj-R^2^ values offer more precise assessments of the model fit. The lower AICc value and higher R^2^ and Adj-R^2^ values indicate a superior fit.

**Figure 4:**
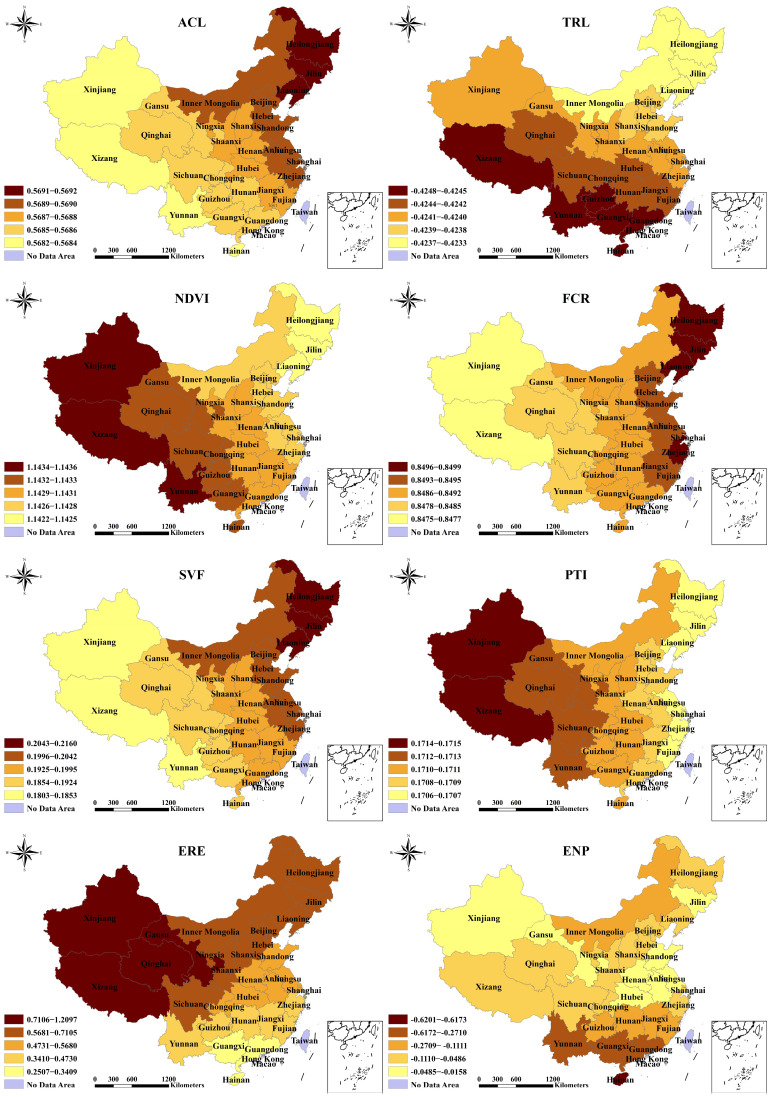
Spatial characteristics of statistically significant coefficients in MGWR 2016–2020.

The coefficients’ statistical results are presented in Table 3 and Table 4. Each explanatory variable’s coefficient was classified into five levels based on the natural breakpoint approach (Figure 4). In the MGWR model, the coefficient of ACL is positive and statistically significant at the 1% level. The coefficient varies between 0.5682 and 0.5692, showing a decline from the northeast to the southwest of the country. For the TRL, the coefficient is negative and statistically significant at the 1% level, with values ranging from −0.4233 to −0.4248. Significant relationships mainly occur in the southwestern and southern provinces of China. The coefficients for NDVI and FCR are both positively and significantly correlated at the 1% level. The western regions are more responsive to NDVI changes. The significant impacts of the latter are mainly located in the northeastern and northern regions. The regional differentiation of the SVF coefficient is comparable to that of the ACL, demonstrating a positive and statistically significant relationship at the *p* < 0.1 level, with values ranging from −0.1803 to −0.2160. The PTI has a positive coefficient that spans 0.1706 to 0.1715, which is statistically significant at the *p* < 0.1 level. There is a decreasing tendency observed from the west to the east. For the ERE, the positive coefficient is statistically significant at the 1% level, and there are considerable variations among the provinces, varying from 0.2507 to 1.2097. The western and northern provinces have a greater coefficient than the other provinces. The ENP coefficient is negatively significant at the *p* < 0.1 level, indicating a more obvious negative impact in southern areas. There is no statistically significant difference in the coefficients for GDP and NED.

### 3.3. Clustering and Zoning of Provinces

**Figure 5:**
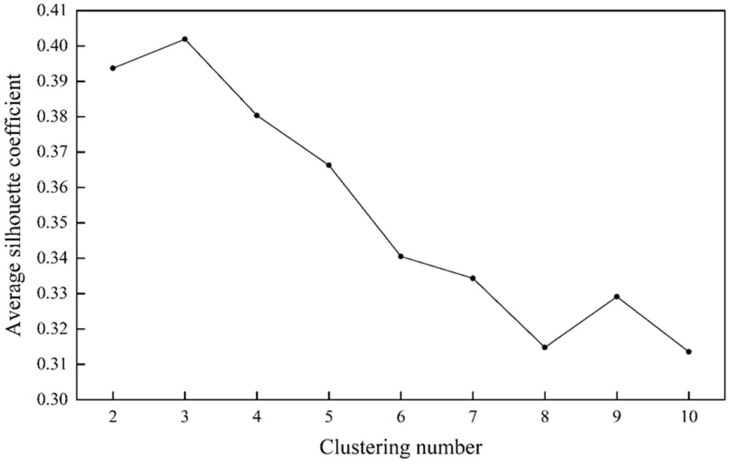
Average silhouette coefficient versus clustering number.

**Figure 6:**
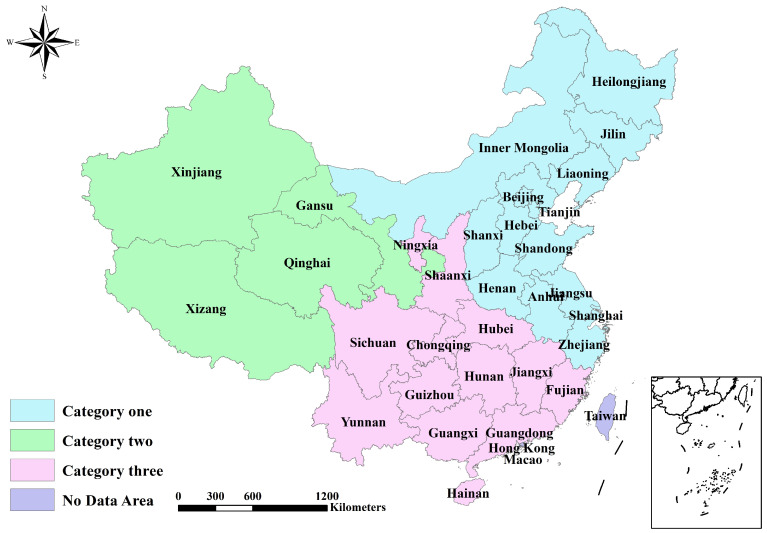
Zoning result of provinces.

The MGWR model generated numerous local coefficients, which can be divided into different categories according to their correlations. The k-means clustering analysis was conducted using the statistically significant coefficients from 31 provinces in China. Given the number of provinces, the zoning number should be less than 10 [44]. The clustering analysis was carried out on 2 to 10 clusters using Python, and the average silhouette coefficient versus the clustering number was plotted, as shown in Figure 5. A higher value represents better clustering quality. The average silhouette coefficient reached its peak at 0.402 when k = 3. Therefore, the optimal clustering observed at k = 3 was used for the zoning, i.e., categorizing 31 provinces into 3 sub-areas (Figure 6).

## 4. Discussion

### 4.1. Analysis of Influencing Factors and Their Spatial Effects

The northeastern part of China has been an important base for heavy industry, while the Beijing–Tianjin–Hebei and Yangtze River Delta urban agglomerations are characterized by developed economies and large populations. Moreover, central heating in northern areas requires a large amount of coal burning. Therefore, these regions are important sources of PM_2.5_ and CO_2_ emissions and are more sensitive to air circulation. Enhanced air circulation facilitates the dispersion of atmospheric pollutants, leading to a notable reduction in pollution levels. The southwestern region is dominated by plateaus and basins, featuring mountainous terrain and significant differences in elevation. The topography of basins causes air pollution to accumulate, making it challenging to disperse and remove. 

Western China experiences arid conditions—characterized by plateaus and deserts, including the Gobi Desert—which significantly restrict vegetation development. Air pollution and carbon reduction are more sensitive to spatial changes in vegetation form, cover degree, health state, and other properties. Hence, a one-unit rise in NDVI results in a more pronounced increase in the reduction index as compared to the eastern region. In some eastern provinces like Shandong and Jiangsu, where FCR and SVF have low national rankings, the advantages of forest resources for carbon reduction and particle removal are not obvious. Therefore, there is a greater potential to enhance the quantity and quality of forest resources to decrease pollution and carbon emissions. 

The tertiary industry has developed rapidly in the eastern provinces. The tertiary sector’s share in the western region is typically lower than in the eastern region, although there is a higher likelihood of the ratio rising. Thus, the evaluation index for PM_2.5_ and CO_2_ reductions is more responsive to increases in PTI in western areas. Climate mitigation and pollution control policies result in geographical variations in technology efficiency and management levels due to the optimization of energy structures and environmental protection investments. In northern and western regions, there is a noticeable beneficial effect, highlighting the necessity to improve the effectiveness of technology and management.

The joint prevention and control of regional air pollution has been a key focus for improving regional air quality since China issued the Action Plan for Air Pollution Prevention and Control in 2013. The Beijing–Tianjin–Hebei, Yangtze River Delta, and Fenhe–Weihe River Plain regions have experienced the most serious air pollution problems. Adjacent cities in these areas are more inclined to collaborate on environmental management strategies and establish targets with consistent levels of strictness. Therefore, the effects of PM_2.5_ emissions from neighboring provinces are less remarkable.

### 4.2. Zoning Features and Policy Implications

Category one is a region characterized by its sensitivity to environmental factors, mostly found in the northern and eastern provinces. Air circulation and forest growth play a significant role in reducing PM_2.5_ and CO_2_ levels in this area. On the one hand, establishing ventilation corridors is crucial. A multi-level ventilation corridor system is constructed in the city based on the prevailing wind direction and existing natural cold sources such as green areas, parks, forests, rivers, and lakes. Building new high-rise structures along these ventilation corridors is strictly prohibited. On the other hand, forest conservation efforts need to be improved. Activity involving greenery and afforestation initiatives should be increased, focusing on reforesting shallow and barren slopes and expanding forested areas in the plains. The use of equipment and infrastructure to improve technical capacity for managing and monitoring forest resources should be expanded. The surveillance, prevention, and management of wildlife disease reservoirs to promote the health of forest ecosystems should also be enhanced.

Category two denotes the integrated sensitivity type, primarily affecting provinces in northwestern China. Variables such as vegetation cover, the proportion of tertiary industry, and emission reduction efficiency have an important effect on air pollution and carbon reductions in these provinces. Firstly, afforestation, returning farmland to forests, returning pasture to grassland, and other greening efforts will be consistently implemented. Secondly, this category focuses on developing tourism, culture, exhibition, and other industries based on available resources and comparative advantages. It also aims to strengthen logistics, commerce, finance, and information services to enhance the tertiary industry’s development and competitiveness. Thirdly, the region should accelerate the transition of energy systems and enhance the development of clean energy. Furthermore, enterprises should be incentivized to enhance reduction efficiency through strategies including process improvement, energy substitution, energy saving, and a more thorough approach to treatment.

Category three is classified as a passive influence and is mainly located in the southern and southwestern provinces. This group shows better a performance in PM_2.5_ and CO_2_ reduction, and the influence of topographical relief and PM_2.5_ emissions from neighboring provinces is relatively greater. However, these provinces find it challenging to alter or adapt to these pressures by changing themselves. Therefore, regional joint prevention and control is necessary to establish a unified planning, monitoring, supervision, and evaluation mechanism among provinces. This involves categorizing areas into key control zones and general control zones based on geographic features, socioeconomic development levels, atmospheric pollution degrees, city distributions, and atmospheric pollutant transport patterns. Targeted pollution prevention and control strategies can then be developed accordingly.

### 4.3. Research Limitations and Future Prospects

The data used in this study were collected at the provincial level and represent each province’s average. In provinces with vast land areas like Inner Mongolia, specific variables like ENP might either enhance or diminish their impacts on other provinces, thereby causing a bias in the regression outcomes. The influencing factors selected are not comprehensive enough. For instance, the ERE indicator should also consider the emission reduction efficiencies of transportation and industrial end-of-pipe measures. In addition, economic policies such as environmental taxes and carbon emissions trading were not included as influencing factors due to data availability.

A city is characterized by high-density human socioeconomic activities, and is the primary source of air pollutants and CO_2_ emissions. Consequently, a city is the most critical entity in effectively managing local air pollutants and CO_2_ emissions, as well as the solution to the issues associated with air pollution and climate change. In further studies, detailed data will be gathered for research on an urban scale. Reduction measures will be refined, such as upgrading industrial boilers, promoting clean fuels in the residential sector, phasing out yellow-label and old vehicles, revising economic policies, etc. This may offer valuable insights into the regional variations in the driving effects of synergistic reduction, as well as insights into appropriate strategies for the co-control of local air pollutants and CO_2_.

## 5. Conclusions

In this study, the reduction indexes of PM_2.5_ and CO_2_ were calculated by carefully considering their reduction bases, reduction efforts, and reduction stabilities. Then, the synergistic reduction effect was evaluated by an exponential function with the PM_2.5_ reduction index as the base and the CO_2_ reduction index as the exponent. The results indicate that, except for the southeastern and southwestern provinces, the remaining provinces are ineffective in synergizing PM_2.5_ and CO_2_ reductions. Next, the elements concerning the synergistic reduction effect were analyzed based on natural settings (air circulation, topography, and vegetation), socioeconomic conditions (economic level and emission reduction efficiency), and external emission impacts. The MGWR model was utilized to examine the directions and magnitudes of the impacts of various influencing factors. The results demonstrate that air circulation, vegetation, tertiary industry ratio, and emission reduction efficiency are major impact indicators that have a positive effect. The topography and the emissions from neighboring provinces have a statistically significant negative impact. The varying degrees of influence from different factors exhibit a spatial distribution trend characterized by high-high aggregation and low-low aggregation. Finally, the k-means clustering method was applied in order to identify the similarities and differences between the coefficients of influencing factors across the 31 provinces. A silhouette coefficient was created to assess the quality of clustering and identify a suitable number of clusters. The results prove that the highest average silhouette coefficient occurs when k = 3, indicating that the most ideal clustering number is 3. Thus, the 31 provincial administrative regions were divided into three categories. Following this, suggestions on the corresponding categories have been proposed, to provide a scientific reference to the synergistic reduction of PM_2.5_ and CO_2_. The relevant findings can clarify the direction of the synergistic management of air pollution and carbon emissions across different regions, in light of their natural environmental characteristics and socioeconomic development conditions. On this basis, more scientific and reasonable policies and measures for synergistic reduction can be formulated to achieve optimal allocation and efficient utilization of resources in all aspects.

## Figures and Tables

**Table 1:** Evaluation results of reduction indexes of 31 provinces from 2016 to 2020.

Province	$I_{R\_PM_{2.5}}$	$I_{R\_CO_{2}}$	*EIPC*
Beijing	0.6605	0.4327	0.7904
Tianjin	0.5300	0.3364	0.6562
Hebei	0.6113	0.1506	0.6584
Shanxi	0.3606	0.0936	0.3967
Inner Mongolia	0.6098	−0.0811	0.5858
Liaoning	0.3454	0.1966	0.4256
Jilin	0.5822	0.2624	0.6710
Heilongjiang	0.5740	0.2189	0.6482
Shanghai	0.6794	0.3848	0.7883
Jiangsu	0.5363	0.3307	0.6590
Zhejiang	0.7799	0.3532	0.8515
Anhui	0.3954	0.2522	0.4997
Fujian	0.8724	0.3249	0.9120
Jiangxi	0.6252	0.2989	0.7194
Shandong	0.3494	0.2634	0.4610
Henan	0.4785	0.3177	0.6047
Hubei	0.5522	0.3300	0.6717
Hunan	0.4253	0.3485	0.5729
Guangdong	0.8002	0.3674	0.8685
Guangxi	0.6613	0.2397	0.7302
Hainan	0.9820	0.3174	0.9877
Chongqing	0.5416	0.3533	0.6726
Sichuan	0.6730	0.3531	0.7740
Guizhou	0.7849	0.2572	0.8353
Yunnan	0.7389	0.2510	0.7972
Xizang	0.9999	0.3347	0.9999
Shaanxi	0.4152	0.2403	0.5129
Gansu	0.6817	0.1421	0.7198
Qinghai	0.8453	0.2822	0.8863
Ningxia	0.3973	−0.3884	0.2776
Xinjiang	0.0005	0.0293	0.0006

**Table 2:** Model diagnostics for OLS, GWR, and MGWR.

Diagnostic Information	OLS	GWR	MGWR
AICc	82.605	82.390	76.671
R^2^	0.778	0.807	0.928
Adj. R^2^	0.667	0.684	0.857

**Table 3:** Descriptive statistics of coefficients in the MGWR model.

Variable	Maximum	Minimum	Mean	Standard Deviation
ACL	0.5692	0.5682	0.5686	0.0002
TRL	−0.4233	−0.4248	−0.4241	0.0003
NDVI	1.1436	1.1422	1.1429	0.0003
FCR	0.8499	0.8475	0.8490	0.0005
SVF	0.2160	0.1803	0.1972	0.0083
GDP	−0.1266	−0.1290	−0.1279	0.0006
PTI	0.1715	0.1706	0.1709	0.0001
ERE	1.2097	0.2507	0.5887	0.2110
ENP	−0.0158	−0.6201	−0.1100	0.1231
NED	0.1603	0.1592	0.1598	0.0002

**Table 4:** Coefficients of explanatory variables for OLS, GWR, and MGWR.

Variable	OLS		GWR		MGWR	
	β	*p*-Value	β (Mean)	*p*-Value (Mean)	β (Mean)	*p*-Value (Mean)
ACL	0.398	0.070 ***	0.405	0.062 ***	0.569	0.001 *
TRL	−0.371	0.044 **	−0.370	0.043 **	−0.424	0.001 *
NDVI	0.961	0.008 *	0.967	0.009 *	1.143	0.000 *
FCR	0.880	0.003 *	0.873	0.004 *	0.849	0.000 *
SVF	0.277	0.054 ***	0.273	0.056 ***	0.197	0.073 ***
GDP	−0.137	0.434	−0.133	0.430	−0.128	0.281
PTI	0.196	0.147	0.191	0.148	0.171	0.062 ***
ERE	0.786	0.000 *	0.762	0.000 *	0.589	0.005 *
ENP	−0.041	0.095 ***	−0.032	0.080 ***	−0.061	0.058 ***
NED	0.180	0.356	0.177	0.348	0.160	0.255

* indicates a statistically significant *p*-value (*p* < 0.01); ** indicates a statistically significant *p*-value (*p* < 0.05); *** indicates a statistically significant *p*-value (*p* < 0.1).

## Data Availability

The raw data supporting the conclusions of this article will be made available by the authors on request.
